# Supplementary material for: Accumulation of unacetylatable Snf2p at the INO1 promoter is detrimental to remodeler recycling supply for CUP1 induction
Source: PLoS One. 2020 Mar 25;15(3):e0230572. doi: 10.1371/journal.pone.0230572 (PMC7094851; doi:10.1371/journal.pone.0230572)
Supplement: S1 File — (DOCX) [file pone.0230572.s001.docx]

**Chromatin Immunoprecipitation (ChIP)**

Crosslinking Cells:

Cells were grown at 30˚C from a single colony in 6ml of appropriate media, depending upon the yeast strain. Cells were then transferred to 394ml of the same media. Once cells reached an optical density of approximately A_600nm_ 0.8 (mid-logarithmic phase), they were divided in half and pelleted. One pellet was washed twice with inducing media (0μM inositol) and the second pellet was washed twice with repressing media (100μM inositol). Each pellet was then resuspended in 200mL of the appropriate media, either inducing or repressing, and incubated for 2 hours at 30˚C, 300 rpm. Each flask of cells was then crosslinked with 5.4ml of Formaldehyde for 35 minutes at 125 rpm. Each flask of cells was then subsequently quenched with 32.7ml of 2.5 M Glycine for 5 minutes at 90 rpm. Cells were then pelleted and washed twice with Cell Wash Buffer.

Cell Lysate Preparation and Chromatin Isolation

Cell pellets were resuspended in 400μl of +PMSF Lysis Buffer (50 mM Tris-HCl pH 7.5, 150 mM NaCl, 1 mM EDTA, 1% Triton X-100) containing 1.5 μM trichostatin A (WAKO)(added as 1.5 mM solution in ethanol), 0.2mM phenylmethylsulfonylfluroride (Sigma Cat#78830) and 0.4 μl of Protease Inhibitor Cocktail Set III (CalBioChem Cat#539134)(added as 100 mM AEBSF, 80 μM Aprotinin, 5 mM Bestatin, 1.5 mM E-64 Protease Inhibitor, 2 mM leupeptin and 1 mM pepstatin A). This cell suspension was then slowly dripped onto 0.45g of acid washed glass beads (0.5mm diameter Sigma, Cat: G8772-500G) in microcentrifuge tube. Each sample was then vortexed in a 4˚C cold room for 20 minutes and spun down at 2K for 10 seconds. The supernatant was then transferred to a new tube and was centrifuged for 15 minutes at 13K in the 4˚C cold room. The supernatant was now discarded and the pellet was resuspended in 1ml ice cold –PMSF Lysis Buffer (same as above but without PMSF). Each sample was then sonicated in 10 second intervals for 1 minute and spun down at 2K for 1 minute at 4˚C. The supernatant was collected and stored at -80˚C.

Quantification (Input DNA Preparation)

10μl of cell lysate was combined with 390μl elution buffer (1% SDS; 0.1M NaHCO_3_) and 16μl 5M NaCl. Samples were incubated in a 65˚C water bath for 6 hours, then 8μl 0.5M EDTA, 16μl 1M Tris-HCl, pH6.5, and 2μl Proteinase K were added and samples were moved to 45˚C for 2 hours. Phenol/chloroform extraction was then performed and the A_260_/A_280_ ratio was determined by UV spectrophotometer analysis with 2μl of sample and 68μl water. DNA concentration was calculated as A_260_*50μg/ml*35 dilution factor.

Immunoprecipitation

11μg of cell lysate was diluted 10 fold in ChIP dilution buffer (0.01% SDS; 1.1% Triton X-100; 1.2mM EDTA; 16.7mM Tris-HCl, pH8; 167mM NaCl) with 1μl TSA and 1μl protease inhibitor cocktail. 35μl of Protein A agarose slurry was then added and samples were precleared at 4˚C for 1 hour with gentle rotations.

Samples were then pelleted at 2K for 10 seconds. Supernatant was collected into a new tube, the appropriate antibody was added (Table S1), and samples were left at 4˚C for overnight rotation. The next morning, 60μl of Protein A agarose slurry was added to each tube and all samples remained rotating at 4˚C for an additional hour. Samples were then pelleted at 2K for 10 seconds and each pellet was then washed with Low Salt Buffer (0.1% SDS; 1% Triton X-100; 2mM EDTA; 20mM Tris-HCl, pH8; 150mM NaCl), High Salt Buffer (0.1% SDS; 1% Triton X-100; 2mM EDTA; 20mM Tris-HCl, pH8; 500mM NaCl), LiCl Buffer (0.25M LiCl; 1mM EDTA; 10mM Tris-HCl, pH8; 1% Triton X-100; 0.1% Deoxycholic Acid), and two rounds of 1X TE Buffer (10mM Tris-HCl; 1mM EDTA). After washes, protein-DNA complexes were then eluted from the resin with two 15 minute incubations gently rotating at room temperature with 250μl of elution buffer each time. The supernatant from each elution was collected, 16μl of NaCl was added, and samples were reverse cross-linked at 65˚C for 6 hours. 8μl 0.5M EDTA, 16μl 1M Tris-HCl, pH6.5, and 2μl Proteinase K were added and samples were moved to 45˚C for 2 hours. Phenol/chloroform extraction was then performed and samples were stored for qPCR analysis.
