# Supplementary material for: Accumulation of unacetylatable Snf2p at the INO1 promoter is detrimental to remodeler recycling supply for CUP1 induction
Source: PLoS One. 2020 Mar 25;15(3):e0230572. doi: 10.1371/journal.pone.0230572 (PMC7094851; doi:10.1371/journal.pone.0230572)
Supplement: S1 Table — (DOCX) [file pone.0230572.s003.docx]

**Table S1. Antibodies used for ChIP experiments**

| **Antibody** | **Manufacturer** | **Catalog Number** |
| --- | --- | --- |
| α-acH3 | Millipore | 06-599 |
| α-acH4 | Millipore | 06-866 |
| α-arp8 | Abcam | 12098-100 |
| α-esa1 | Abcam | 4466-100 |
| α-FLAG | Sigma | 7425 |
| α-gcn5 | Santa Cruz | y-300 |
| α-H3 | Abcam | 1791 |
| α-H4 | Abcam | 10158 |
| α-pol II | Millipore | 05-623 |
