# Supplementary material for: Accumulation of unacetylatable Snf2p at the INO1 promoter is detrimental to remodeler recycling supply for CUP1 induction
Source: PLoS One. 2020 Mar 25;15(3):e0230572. doi: 10.1371/journal.pone.0230572 (PMC7094851; doi:10.1371/journal.pone.0230572)
Supplement: S2 Table — (DOCX) [file pone.0230572.s004.docx]

**Table S2. Primer sequences for Reverse Transcriptase PCR**

| Name | Sequence (5’ to 3’) |
| --- | --- |
| *INO1* Forward | CCATGGTTAGCCCAAACGA |
| *INO1* Reverse | GCCTTCAAGCGTTGTTGCA |
| *CUP1* Forward | AAGGTCATGAGTGCCAATGC |
| *CUP1* Reverse | ATTTCCCAGAGCAGCATGAC |
| *ACT1* Forward | CCAAGCCGTTTTGTCCTTGT |
| *ACT1* Reverse | ACCGGCCAAATCGATTCTC |
