# Supplementary material for: Accumulation of unacetylatable Snf2p at the INO1 promoter is detrimental to remodeler recycling supply for CUP1 induction
Source: PLoS One. 2020 Mar 25;15(3):e0230572. doi: 10.1371/journal.pone.0230572 (PMC7094851; doi:10.1371/journal.pone.0230572)
Supplement: S3 Table — (DOCX) [file pone.0230572.s005.docx]

**Table S3. Primer and Probe sequences for Real-Time qPCR**

| Name | Sequence (5’ to 3’) |
| --- | --- |
| *INO1* Forward | GAAATATGCGGAGGCCAAGTAT |
| *INO1* Reverse | ACGCAGAGGTGCGCTTTCT |
| *INO1* Probe | [6FAM]CGCTTCGGCGGCT[BHQ1a-Q] |
| *CUP1* *UAS* Forward | AAAAGACATTTTTGCTGTCAGTCACT |
| *CUP1* *UAS* Reverse | GGAACGGTTCAGCGGAAA |
| *CUP* *UAS* Probe | [6FAM] AAGAGATTCTTTTGCTGGCAT [BHQ1a-Q] |
| *CUP1* *TATA* Forward | AAAGACTACCAACGCAATATGGATT |
| *CUP1* *TATA* Reverse | GCAATTGATACAAGACAAGGAGTTA |
| *CUP* *TATA* Probe | [6FAM] TCAGAATCATATAAAAGAGAAGC [BHQ1a-Q] |
