# Supplementary material for: Accumulation of unacetylatable Snf2p at the INO1 promoter is detrimental to remodeler recycling supply for CUP1 induction
Source: PLoS One. 2020 Mar 25;15(3):e0230572. doi: 10.1371/journal.pone.0230572 (PMC7094851; doi:10.1371/journal.pone.0230572)
Supplement: Attachment — Submitted filename: Review of Shen etal Plos One.docx [file pone.0230572.s006.docx]

The article by Shen et al, is an elegant study on the role of Snf2 acetylation in regulating expression of genes. It carries forward studies from the Workman lab and provides a further detailed basis for how acetylation of Snf2 can affect gene expression by regulating other chromatin remodelers and suppress gene expression. The authors show, as would be predicted, that loss of Snf2 acetylation delays release of Snf2 from gene promoters. Very interestingly they show that Snf2 acetylation also affects release of INO80 from gene promoters. Additionally, the authors show that failure to release and recycle Snf2, decreases CUP1 expression. This is a well done study and should be accepted for publication after the few comments below are addressed.

Specific comments:

1) Can the authors show that loss of CUP1 expression in the unacetylable Snf2 is due to failure of Snf2 to be recruited to CUP1 promoter by ChIP?

2) Loss of Snf2 acetylation primarily affects release of Snf2. If the authors first induce INO1 and then suppress INO1 by adding back Inositol, does this affect Snf2, Ino80, PolII occupancy at INO1 and affect gene expression and histone levels?
