# Supplementary material for: Accumulation of unacetylatable Snf2p at the INO1 promoter is detrimental to remodeler recycling supply for CUP1 induction
Source: PLoS One. 2020 Mar 25;15(3):e0230572. doi: 10.1371/journal.pone.0230572 (PMC7094851; doi:10.1371/journal.pone.0230572)
Supplement: Attachment — Submitted filename: Response to Reviewers.docx [file pone.0230572.s007.docx]

Response to Reviewers:

Reviewer #1:

Point#1

To rule out the possibility that low *CUP1* mRNA levels is not the stability of *CUP1* mRNA but instead transcription that is adversely affected in the unAcSnf2p mutant, we have performed the RNA polymerase II-ChIP on *CUP1* promoter region as directed by the reviewer. We have demonstrated that *CUP1* expression in unAcSnf2p strain is significantly repressed under *INO1* inducing conditions, and this is due to the inability to recruit RNA polymerase. The result has been shown in Figure 6C.

Point #2

To show that the loss of *CUP1* expression in the unAcSnf2p strain (Snf2p is unacetylable in this strain) is due to the failure of Snf2p recruitment to *CUP1* promoter, we have performed the Snf2p IP. Our results showed that Snf2p is not recruited to unAcSnf2 strain’s *CUP1* promoter under *INO1* inducing conditions. This confirmed that the unacetylable Snf2p accumulated at *INO1* promoter under *INO1* inducing conditions and this unacetylable Snf2p accumulation resulted in the failure of Snf2p recruitment at *CUP1* promoter when *CUP1* is under inducing conditions. The result has been shown in Figure 6D.

Point #3

We revised the Discussion section by removing some paragraphs that are redundant to the Result section, so that the revised manuscript is more concise and clear. Please see the marked-up copy for the detail.

Reviewer #2:

Point #1

This comment is exactly the same as the Reviewer #1’s Point #2 comment and we have responded the comment. Please see the response (2) under Reviewer #1.

Point #2

Since the loss of Snf2p acetylation primarily affects the release of Snf2p, we also performed the switching *INO1* expression conditions experiment as directed by the reviewer. Our results showed that *INO1* expression, nucleosome density, recruitment of remodelers, and remodeling activities are independent of Snf2p acetylation when switching from inducing conditions to repressing conditions. The results has been presented in Figure 4.
